# Monte Carlo Dropout for Uncertainty‐Aware Alzheimer's Disease Classification Using Transformer Models on Whole‐Genome Sequencing Data

**DOI:** 10.1002/alz70856_105659

**Published:** 2026-01-07

**Authors:** Taeho Jo

**Affiliations:** ^1^ Department of Radiology and Imaging Sciences, Indiana University School of Medicine, Indianapolis, IN, USA

## Abstract

**Background:**

Alzheimer's disease (AD) is a progressive neurodegenerative disorder marked by cognitive decline and memory impairment. Early and accurate detection is critical for clinical intervention. While machine and deep learning approaches have been widely used to predict AD progression, recent work emphasizes quantifying predictive uncertainty in high‐stakes medical contexts. However, many studies focus on limited genetic regions (e.g., APOE), highlighting the need for broader whole‐genome sequencing (WGS) analyses.

**Method:**

We obtained 1,050 WGS datasets (443 cognitively normal, 607 AD‐diagnosed) from ADNI, ADNI‐WGS‐2, and ADSP‐FUS1‐ADNI‐WGS‐2. SNPs were extracted from a region containing the APOE gene on chromosome 19, then divided into fixed‐size windows (“tokens”) for a Transformer‐based classification model. Monte Carlo (MC) Dropout was applied during training and inference to enable multiple forward passes, providing predictive variance. Models with and without MC Dropout were compared using accuracy (ACC), area under the curve (AUC), F1 score, sensitivity, specificity, expected calibration error (ECE), and reliability diagrams. Predictions were further stratified into “Uncertain” (top 25% variance) and “Certain” (bottom 75%) to examine accuracy differences.

**Result:**

Stratification by predictive variance revealed that the Uncertain group, with 53 samples, had an accuracy of 0.5472, while the Certain group, with 157 samples, had 0.6497. This indicates that Monte Carlo Dropout–derived variance effectively captures higher uncertainty. The Monte Carlo Dropout model showed a slight increase in accuracy from 0.6143 to 0.6238 and in area under the curve from 0.6644 to 0.6832 compared with baseline, but calibration worsened when the expected calibration error rose from 0.1024 to 0.1858. Sensitivity and specificity shifted from 0.7417 to 0.6833 and from 0.4444 to 0.5444, while the F1 score decreased from 0.6873 to 0.6749.

**Conclusion:**

These findings demonstrate the feasibility of using MC Dropout within a Transformer‐based WGS framework to estimate predictive uncertainty in AD classification. Variance‐based stratification effectively flagged samples with lower confidence. However, MC Dropout did not consistently improve calibration or overall accuracy, indicating a need for refining dropout settings, Transformer hyperparameters, and potentially Bayesian time‐series methods. Future work should also explore complementary approaches to balance classification accuracy with reliable uncertainty estimates.

